# Computed Tomography Findings of Ruptured Hepatic Hydatid Cyst into the Pericardial Space: A Case Report

**Published:** 2019

**Authors:** Sercan ÖZKAÇMAZ

**Affiliations:** Department of Radiology, Faculty of Medicine, Kırşehir Ahi Evran University, Kırşehir, Turkey

**Keywords:** Hydatidosis, Rupture, Computed tomography, Pericardial effusion

## Abstract

Hydatidosis, is a parasitic infestation caused by *Echinococcus granulosus*. Although the disease most commonly affects liver and lungs, almost all organ and tissue involvements are documented. Rupture into pericardial space which may lead to pericardial effusion, pericarditis and pericardial tamponade, can be seen especially in the patients with cardiac hydatidosis. But rupture of a hepatic hydatid cyst into the pericardial space through a transdiaphragmatic fistula is very rare. In this report, we present imaging findings of a type III hepatic hydatid cyst lesion which ruptured spontaneously into pericardial space and caused pericardial effusion.

## Introductıon

Hydatidosis, is a parasitic infestation which is caused by *Echinococcus granulosus* which can involve any organ or tissue. Although the most common affected sites are liver and lungs, cardiac involvement can be also reported. An important complication of cardiac involvement is the rupture of the cyst into pericardial space which leads to pericardial effusion. But rarely pericardial effusion occurs secondary to rupture of a hepatic hydatid cyst through a fistula tract between the cyst and pericardial space (1).

In this report, we present Computed Tomography (CT) findings of a case with type III hepatic hydatid cyst which ruptured into the pericardial space and caused pericardial effusion

## Case Report

**Fig. 1:**
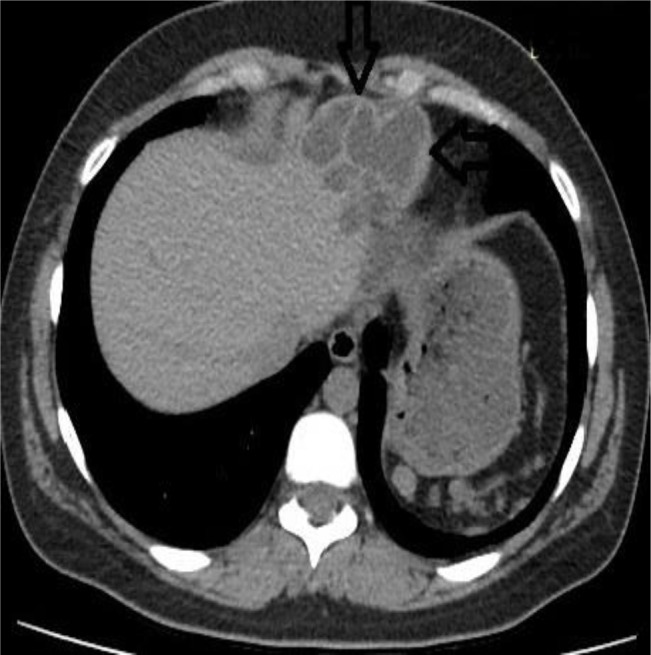
Axial CT image of the patient at first admission, shows a non-complicated type III hydatid cyst in left hepatic lobe (Arrow)

**Fig. 2:**
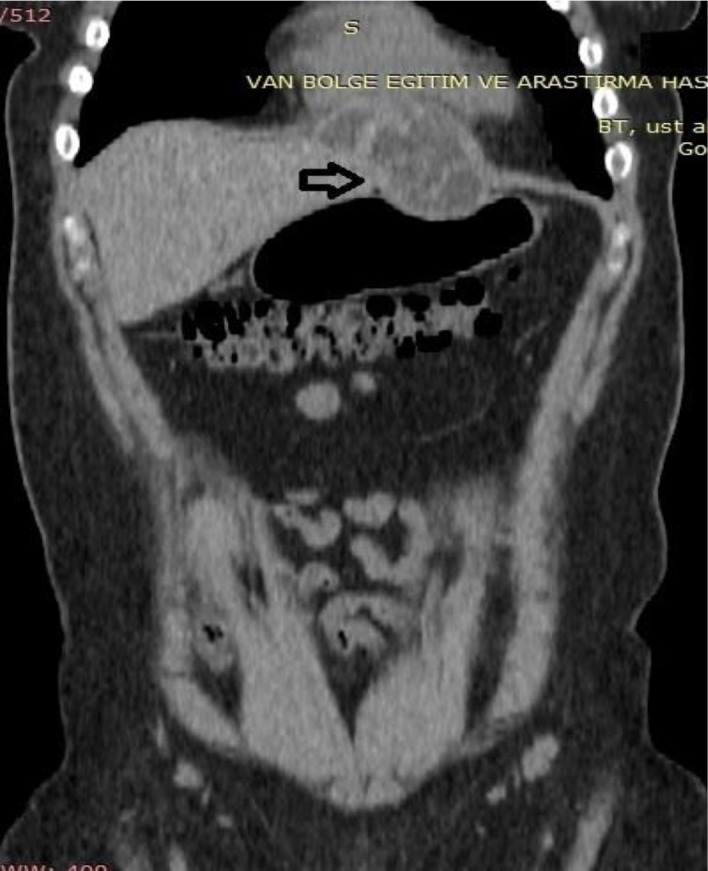
A coronal reformatted CT image at first admission shows type III hydatid cyst in left hepatic lobe which causes elevation of diaphragm and cardiac indentation.(Arrow)

A 30 yr old female, admitted to Gastroenterology Clinic with a complaint of right upper quadrant pain. Physical examination revealed a palpable mass in the epigastric area. Ultra-sound demonstrated subdiaphragmatic thick walled multiloculated hepatic cyst consistent with type III hydatid cyst in the left hepatic lobe. Also CT imaging well identified the cystic lesion which extended superiorly and led to elevation of diaphragm segment that is just below pericardium (Fig. 1–2). Laboratory findings was unremarkable except a mild eosinophilia (eosinophil count was 900/ml). Serum samples of the patient were found to be positive for *Echinococcus* antibodies by ELISA. With the radiological and laboratory findings, a diagnosis of hepatic hydatid disease was made and oral albendazole treatment was started.

**Fig. 3:**
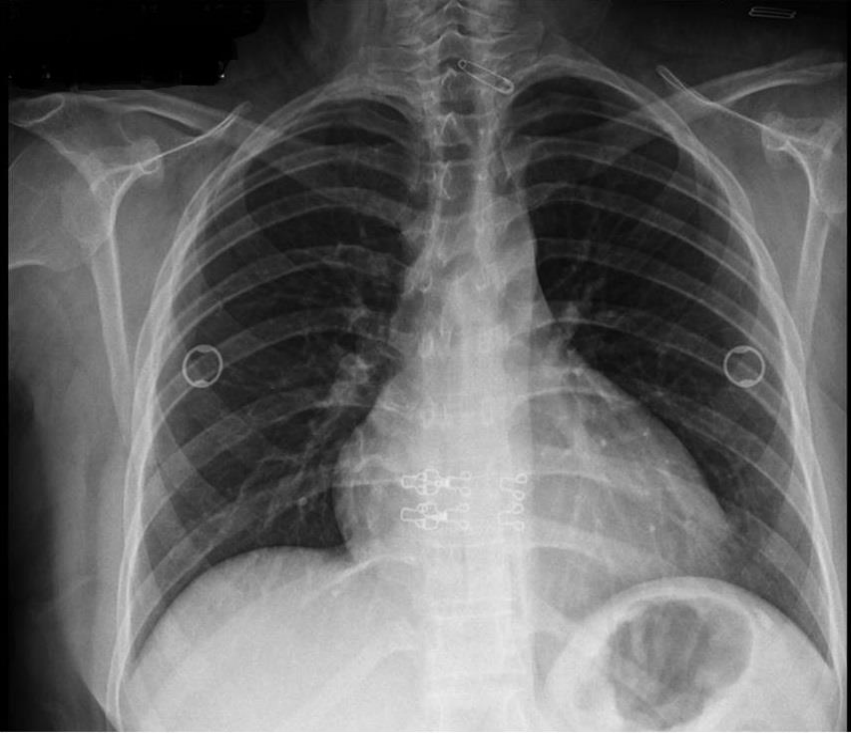
A PA chest radiograph at the second admission demonstrates increased cardiothoracic ratio which suggests cardiomegaly or pericardial effusion

**Fig. 4:**
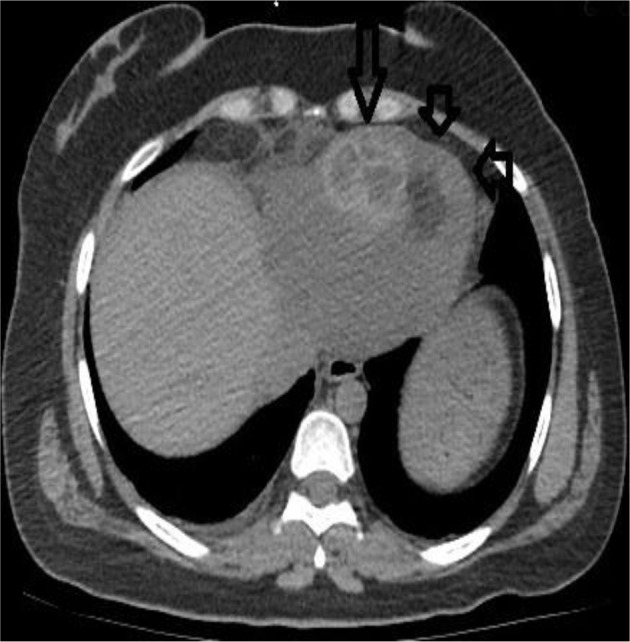
Axial CT image at the second admission demonstrates pericardial effusion (short arrows) associated with hydatid lesion (Long arrow)

**Fig. 5:**
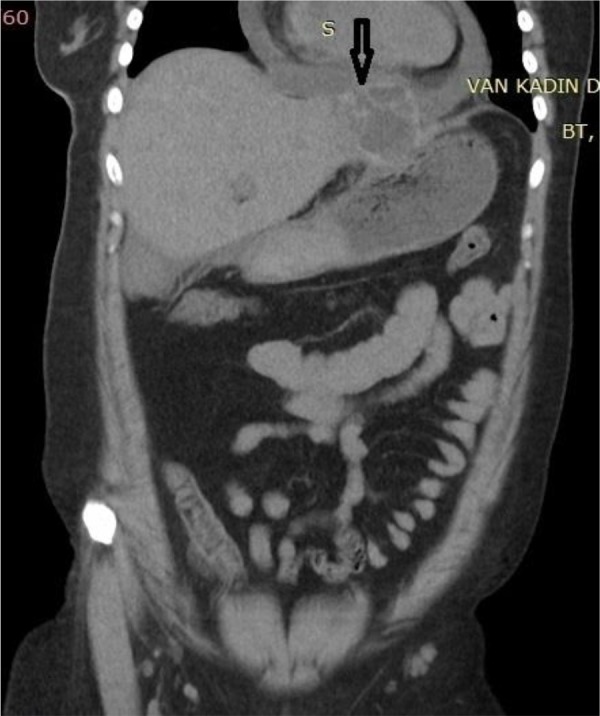
A coronal reformatted CT image at second admission detects a diaphragmatic defect between cyst and pericardial space which is consistent with a trans diaphragmatic fistula (Arrow)

**Fig. 6:**
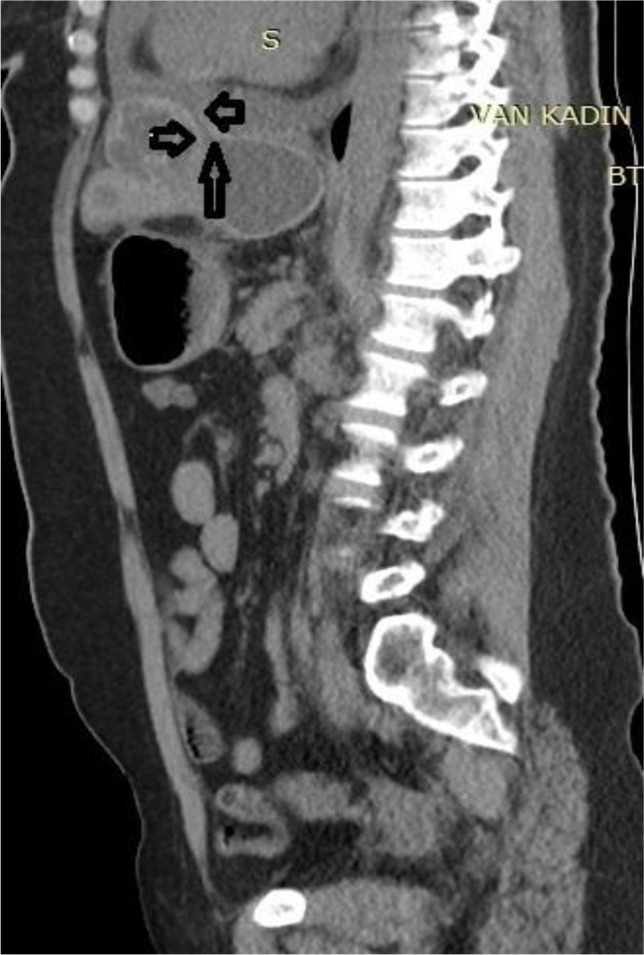
A sagittal reformatted image at second admission shows irregularity of the cyst wall (long arrow) and the fistula tract between cyst and pericardial space (short arrows)

Eighteen months later, she readmitted with epigastric/chest pain and dyspnea for a duration of 5 days. Computed tomography demonstrated pericardial effusion (2 cm in thickness) and a small diaphragmatic defect between the cyst and pericardial cavity which is consistent with a fistula tract between cystic lesion and pericardial space (Fig. 3–6). Partially pericardiectomy, repair of diaphragmatic defect and evacuation of the cyst were performed.

## Discussion

Hydatidosis is a systemic zoonosis which most common affects liver and lungs. However almost all organ or tissue involvements were reported. Cardiac involvement of hydatidosis is rare which includes left ventricle, right ventricle, pericardium, pulmonary artery, left atrial appendage and interventricular septum involvements. Rupture of these cysts into pericardial space can lead to pericarditis, pericardial effusion or pericardial tamponade (2).

Most common complications of hepatic hydatid cysts are rupture into peritoneal cavity which leads to peritonitis or dissemination, intrabiliary rupture, contained rupture, rupture into hepatic subcapsular space, transdiapraghmatic thoracic rupture, secondary bacterial infections, and abscess formation. The frequent imaging findings on CT which suggest transdiapraghmatic thoracic rupture are pleural effusion, atelectasia and lung consolidation which result from accumulation of hydatid fluid into the thorax (3).

Although the most of the cases with ruptured cyst hydatid lesions into pericardial space occur secondary to primary cardiac hydatid cysts, very rarely hepatic hydatid cysts which ruptured into pericardial space through a transdiapraghmatic fistula are reported. These ruptures may lead to pericardial effusion, pericarditis and pericardial tamponed. Transthoracic echocardiography, CT and Magnetic Resonance Imagıng are the most used imaging methods to detect such cardiac hydatic disease. Hatemi et al. reported a case with a grade III hepatic hydatic cyst which ruptured into pericardial space and led to constrictive pericarditis. They presented the CT findings of pericardial effusion and thickened pericardium of their patient (1).

Herrero et al. presented CT findings of a case with a multivesicular hydatid cyst located in liver segments 2 and 3 which ruptured into pericardial space and led to a large global pericardial effusion. After two weeks of albendazole treatment, their patient underwent cystopericystectomi and evacuation of pericardial effusion (4).

Yağmur et al reported CT and ultrasound findings a patient with cardiac tamponade due to rupture of a type III hepatic hydatid cyst into pericardial space. After evacuation of pericardial and cyst contents, the empty cavity was cleaned and sterilized with povidone-iodine 10% solution. Following partially cystectomy, on postoperative day 20,the chest xray demonstrated a normal cardiothoracic ratio (5).

Ahuja et al presented a 10 yr old male with a large hepatic hydatic cyst ruptured into subdiaphragmatic -pericardial space and also caused pericardial effusion and right-sided reactionary pleural effusion. Following aspiration and enucleation of the cyst oral albendazole treatment was started. On follow-up one month, imaging methods showed total resolution of pericardial and pleural effusion (6).

In literature with our case we found a total of 5 cases with hepatic cyst hydatid disease which led to pericardial effusion through a transdiaphragmatic fistula with radiological findings. Among of them four ones were located in left hepatic lobe and the other one was located in right hepatic lobe. Three of them occurred in males and two ones occurred in females. Also four of these patients were adults while one patient was child. But in all these cases, cysts ruptured into pericardial space spontaneously without trauma. This findings suggests that chronic process with recurrent compression of neighboring organs such as stomach can contribute to the rupture. The most of the cases (four of five cases) were located in left hepatic lobe suggests a probable such causative relationship between cyst localization and rupture into pericardial space.

Also in these five case, all the ruptured cysts are type III (According to Gharbi classification) cyst hydatid lesions. In literature, no causative reason was suggested for this relationship but this rupture tendency of type III cyst hydatid lesions may result from the resistance of the lesion which is due to septates or semisolid components within the cyst. This structure of type III hydatid cysts may allow the cyst wall to erode diaphragm and cause a fistula formation between the cyst and pericardial space.

In our case a time between the first diagnosis and the complication of pericardial effusion was 18 months. Such relative long duration may also leads to aging of the cyst and degeneration of the cyst wall which can contribute the rupture.

Because of in literature with our patient all the hepatic hydatid diseases associated with rupture into pericardial space are type III cyst hydatid lesions, this type of cysts can be followed closer. And also because of the long-term compression of a distended stomach may cause pericardial rupture tendency of a left subdiaphragmatic cyst hydatid lesion, such located cysts can be followed closer or may be consider as a candidate for surgery.
